# Effect of five hours of mixed exercise on urinary nitrogen excretion in healthy moderate-to-well-trained young adults

**DOI:** 10.3389/fnut.2024.1345922

**Published:** 2024-02-21

**Authors:** Matthieu Clauss, Meike Burkhardt, Sophie Wöber, Bjørn Steen Skålhegg, Jørgen Jensen

**Affiliations:** ^1^Department of Physical Performance, Norwegian School of Sport Sciences, Oslo, Norway; ^2^Department of Sport and Sport Science, University of Freiburg, Freiburg, Germany; ^3^Department of Nutrition, Division for Molecular Nutrition, University of Oslo, Oslo, Norway

**Keywords:** urinary nitrogen excretion, sweat nitrogen excretion, protein metabolism, endurance exercise, 3-methylhistidine excretion

## Abstract

**Introduction:**

Carbohydrates and fats are the primary energy substrates during exercise, but proteins can also contribute. When proteins are degraded in the body, the amino groups are mainly converted to urea and excreted. Therefore, nitrogen excretion has been used as a marker of protein degradation, but a clear conclusion has yet to be reached on the effect of exercise on nitrogen excretion. Thus, we tested whether exercise increases nitrogen excretion.

**Methods:**

Fifteen young, healthy, moderate-to-well-trained participants (4 females, 11 males, VO_2max_ 54.4 ± 1.7 mL·kg^−1^·min^−1^; mean ± SEM) participated in a randomized, balanced cross-over design investigation consisting of 1 day with 5 h of exercise (exercise day, EX) and 1 day with no exercise (control day, CON). The participants recorded their dietary intake the day before from 16:00 and throughout the intervention day. They then repeated these dietary intakes on the second trial day. A standardized lunch was provided on both days. In addition, participants were allowed to consume almost protein-free snacks in EX to ensure the same energy balance during both trial days. Urine was collected throughout the whole testing period, and urinary 3-methylhistidine (3-MH) excretion was measured to examine muscular catabolism. The sweat rate was calculated during the exercise period.

**Results and discussion:**

The urinary nitrogen and 3-MH excretions did not differ significantly between EX and CON (*p* = 0.764 and *p* = 0.953). The sweat rate was 2.55 ± 0.25 L in EX and 0.14 ± 0.15 L in CON (*p* < 0.001), and by estimating sweat nitrogen excretion, total nitrogen excretion was shown to differ with exercise. Our results showed that 5 hours of mixed exercise did not significantly impact urinary nitrogen and 3-MH excretions in healthy moderate-to-well-trained young adults.

## 1 Introduction

The view of proteins as an energy substrate has changed over time. In 1842, Justus von Liebig suggested that protein was the “only true nutrient, providing both the machinery of the body and the fuel for its work” (1). Not long after Liebig’s claim, several studies proved this conclusion wrong (2). Nevertheless, von Liebig’s claim led to many studies combining exercise and the measurement of nitrogen excretion via urine collection during the late 19^th^ century. Many of these studies showed rises in nitrogen excretion after physical work or exercise (see Supplementary Table S1). However, nitrogen excretion was seldom quantified reliably, nor was the data collection accompanied by a standardized diet or a control trial within the same subjects (3–12). This makes the comparison of studies challenging, as nitrogen excretion is highly correlated with protein (nitrogen) intake from food (13). It is important to note that dietary proteins are the only exogenous nitrogen source that can regulate the body’s nitrogen metabolism. In addition, nitrogen excretion decreases as energy intake increases at a fixed nitrogen intake (13). Furthermore, few participants were included in the studies and no specific energy expenditures were calculated during the work/exercise periods. Consequently, no clear conclusion has been reached on the effect of exercise on urinary nitrogen excretion.

Since then, some newer studies have investigated the effect of exercise on urinary nitrogen excretion in more reliable designs. Urine was collected on an exercise day, including a running or cycling exercise, and compared to urine collected on a resting day (14–17). Urinary nitrogen excretion was either significantly higher or tended to be higher during the exercise day versus the control day. However, another study with a similar cycling exercise did not measure any difference in urinary nitrogen excretion between the exercise and the control day (18). Thus, a clear conclusion remains to be reached on the effect of exercise on urinary nitrogen excretion. As nitrogen excretion provides a reasonable estimate of whole-body protein degradation, arriving at a conclusion would allow several practical applications.

During exercise, nitrogen can also be excreted in sweat. However, the amount of sweat nitrogen excreted varies greatly. Some studies have shown as low as ≈50 mg·h^−1^ of nitrogen excreted in sweat (19), some ≈250 mg·h^−1^ (17, 20), some ≈500 mg·h^−1^ (21), and others reported ≈600 mg·h^−1^ (22, 23), and up to ≈1700 mg·h^−1^ (24). Despite the large disparity, most values fall within the same concentration range of approximately 0.4 to 1.2 mg/mL. Differences in temperature and dietary nitrogen intake are the main factors explaining the variance within this range (19, 25). A significant linear relationship exists between nitrogen intake and sweat nitrogen loss at a fixed temperature (20, 25). Therefore, it is possible to estimate sweat nitrogen loss if both factors are known, and the sweat rate is measured.

A further *in-vivo* non-invasive method for quantifying skeletal myofibrillar protein catabolism is the measurement of urinary 3-methylhistidine (3-MH) excretion (26, 27). The amino acid 3-MH is formed by adding a methyl group to specific histidine residues in the peptide chains of actin in all muscles and myosin in white muscle fibers (28–30). About 80% of the excreted 3-MH in adults is derived from actin and 20% from myosin (31). When muscle protein is degraded, 3-MH is neither reused for protein synthesis (32) nor metabolized (33) and is excreted in the urine (34). Because plasma levels of 3-MH are low and renal clearance is high, urinary excretion reliably reflects myofibrillar degradation rates.

Previous studies have shown that when participants cycle to exhaustion, they need to ingest a large quantity of protein to be in nitrogen balance (35–37). This indicates increased nitrogen excretion, but it remains to be proven. Therefore, in this study, participants completed a randomized, balanced cross-over design of two trial days. One day consisted of 5 h of exercise (EX), and the other comprised 5 h of rest and no exercise of any form (CON). Urine samples were collected during the entire testing period. Based on this, we hypothesized that the exercise day would increase urinary nitrogen and 3-MH excretions.

## 2 Materials and methods

### 2.1 Participants

Fifteen young, healthy, moderate-to-well-trained adults (4 women and 11 men) were recruited for the study. Their characteristics are described in Table 1. Participants were informed about the study before providing their written informed consent. In addition, all participants completed a health questionnaire to rule out potential risk factors. The Norwegian School of Sport Sciences’ Ethics Committee (Application 190–170,621) and the Norwegian Centre for Research Data (Reference number 473323) approved the study, which conformed to the standards set by the latest revision of the Declaration of Helsinki.

**Table 1:** Age, anthropometric data, and VO_2max_ in pretests.

Parameter	Women and men (*n* = 15)	Women (*n* = 4)	Men (*n* = 11)
Age (years)	24.4 ± 0.7	23.1 ± 1.2	24.8 ± 0.8
Body weight (kg)	73.6 ± 1.8	69.9 ± 4.7	74.9 ± 1.8
Height (m)	1.78 ± 0.02	1.71 ± 0.03	1.80 ± 0.02^*^
BMI (kg·m^−2^)	23.2 ± 0.4	23.7 ± 0.9	23.0 ± 0.4
Lean mass (kg)	57.1 ± 1.6	48.7 ± 2.0	60.2 ± 1.0^*^
Fat mass (kg)	14.9 ± 1.3	19.8 ± 2.4	13.1 ± 1.2^*^
Fat mass (%)	20.2 ± 1.7	28.0 ± 2.2	17.3 ± 1.3^*^
VO_2max_ (L·min^−1^)	4.0 ± 0.1	3.3 ± 0.3	4.3 ± 0.1^*^
VO_2max_ (mL·kg^−1^·min^−1^)	54.4 ± 1.7	47.5 ± 3.1	57.0 ± 1.4^*^
HR_max_ (beat·min^−1^)	195.1 ± 1.9	197.8 ± 5.2	194.2 ± 1.9

Data are given as average ± SEM. ^*^p < 0.05 compared to women. BMI body mass index; HR_max_ maximum heart rate; VO_2max_ maximum oxygen uptake.

### 2.2 Study design

**Figure 1:**
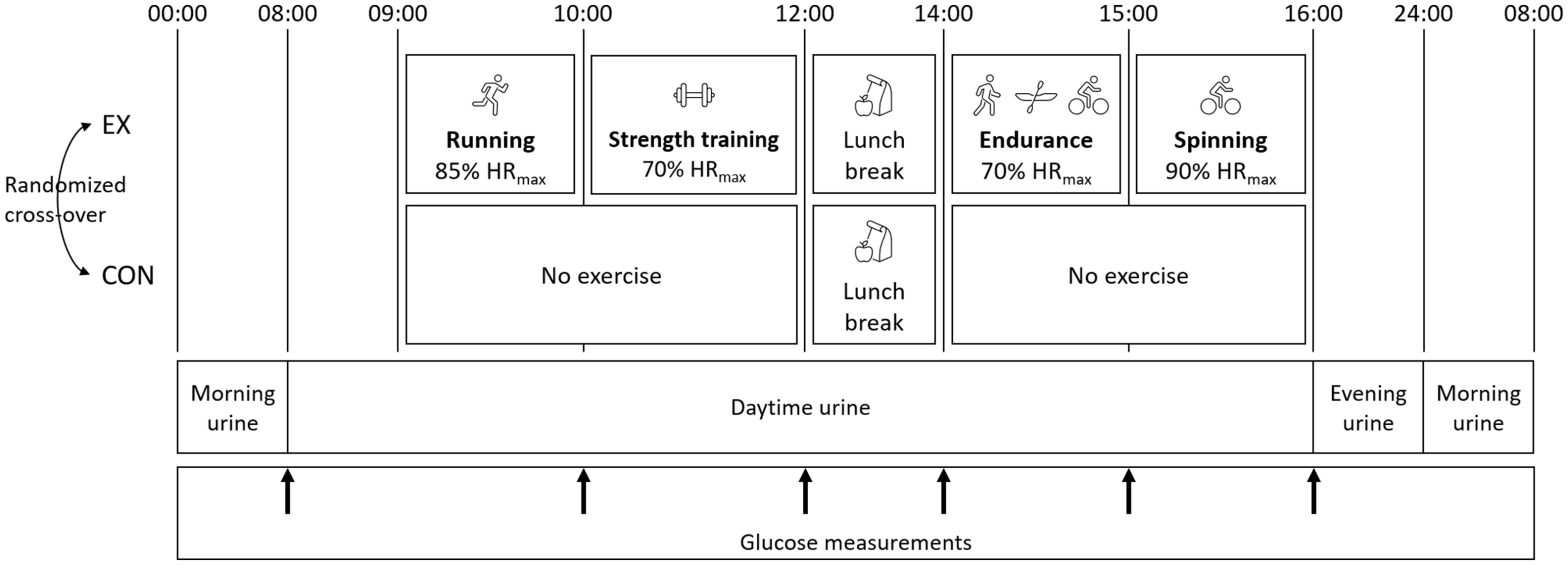
Design of the interventions. The protocol was completed in a randomized, balanced crossover experimental design. The study consisted of two trial days: one exercise (EX) and one control day (CON). During CON, no physical activity was performed during the trial day. The same diet was consumed the day before and during the two trial days, apart from snacks in EX. The urine collection periods are indicated in the figure.

This study was performed in a randomized, balanced cross-over design (Figure 1). The study consisted of two trial days: one exercise (EX) and one control day without physical activity (CON), preceded by a screening day to determine physiological parameters and measure body composition. The same samples were collected in EX and CON. Dietary intakes were identical, except participants were allowed to consume almost protein-free snacks during EX to ensure the same energy balance during the two trial days.

### 2.3 Screening day

#### 2.3.1 Incremental test

Participants performed an incremental test on a cycle ergometer (Lode Excalibur, Lode, Groningen; The Netherlands), during which the relationship between oxygen uptake and heart rate was established. This test was performed in a controlled environment under similar ambient conditions (18–19°C). The saddle and handlebar positions were individually adjusted. The test consisted of 3 to 5 steps of 5 min, with the load increasing by 25 W for each step. The starting load was selected based on the height and body weight of the participant, expected VO_2max_, and previous cycling experience. The pedaling frequency was 75 rpm, which had to be maintained throughout the test. Oxygen uptake was measured online (Oxycon Pro; Jager Instruments, Hoechberg, Germany) over 150 s (from ~2.5–5 min at each intensity level), and capillary blood samples were taken for lactate analysis (Biosen C-Line Lactate analyzer, EKF Diagnostics, United Kingdom). Heart rate (Polar RS800CX, Kempele, Finland) was measured continuously throughout the incremental test. The incremental test was terminated after 5 steps or when the blood lactate concentration exceeded 4 mM. Linear regression analysis from this incremental test was used to establish a relationship between oxygen uptake and heart rate.

#### 2.3.2 Maximum oxygen uptake test

After 10 min of rest following the previous incremental test, maximal oxygen uptake (VO_2max_) was measured using the same equipment. Participants started the VO_2max_ test at the second to last step of the incremental test, and intensity was increased by 25 W steps every 60 s until voluntary exhaustion. The pedaling frequency was 75 rpm. Oxygen uptake was measured continuously, and the mean of the two highest measurements was defined as maximal oxygen uptake (VO_2max_). Heart rate (Polar RS800CX, Kempele, Finland) was measured continuously throughout the maximum oxygen uptake test. The maximum heart rate (HR_max_) was the highest heart rate reached during this test.

#### 2.3.3 Body composition

Body composition was measured using a whole body Dual-Energy-X-ray Absorptiometry (Scanex Medical Systems AS, GE Lunar iDXA, GE Healthcare, program ENCORE 18). Fat, lean, bone (bone mineral content), and total body mass were determined.

### 2.4 Trial days

Participants recorded their dietary intake from 16:00 to bedtime the day before the first trial day and were asked to follow the same diet before the second trial day. No exercise was allowed within the last 24 h before the trial days. Participants were randomly assigned to either EX or CON first. When conducting EX first, a minimum interval of 7 days was set before completing CON to ensure proper recovery. When conducting CON first, a minimum interval of 2 days was set before completing EX.

#### 2.4.1 Exercise day (EX)

During EX, participants performed 5 h of endurance-based exercise: 1 h of running, 2 h of body weight strength circuit training, 1 h of cardio endurance workout, and 1 h of cycling to exhaustion on a stationary spinning bike. The exercise intensity for these exercises was selected by the participants in a certain intensity range to ensure participants could successfully adhere to the prescribed exercise protocol. Monitoring of the intensity was conducted through both the percentage of maximum heart rate (%HR_max_) and the participants’ rating of perceived exertion (RPE).

Participants reported to the laboratory at 8:00. Each participant’s body weight was measured as well as resting blood glucose concentration (HemoCue Glucose 201 RT Analyzer; HemoCue AB, Ängelholm, Sweden). The resting heart rate was measured after 5 min of sitting quietly. Participants then prepared for the exercise.

At 9:00, the participants performed 1 h of outdoor running. Throughout the whole EX, heart rate (Polar RS400, Kempele, Finland) was measured continuously, and the Rate of Perceived Exertion (RPE) (38) was reported at regular intervals. The running exercise was performed at 80–90% HR_max_, with an RPE of 11–16. Capillary blood glucose concentration was measured directly after running.

At 10:00, participants began 2 h of body weight strength circuit training consisting of 6 circuits of four exercises (4 rounds of 30 s intervals, 30 s break; 16 min per circuit) with a 4 min break between each circuit. Circuit 1 consisted of push-ups, crunches, squats, and paddles. Circuit 2 consisted of lunges, sit-ups diagonal, dips, and planks. Circuit 3 involved squat jumps, abs-bug, mountain climber, and shoulder push-ups. Circuit 4 consisted of drop jumps, shoulder press with small weights, Russian twist, and rowing bent forward with small weights. Circuit 5 comprised side lunges, curls with small weights, scissor abs, and swimming abs. Circuit 6 consisted of skipping, step jumps alternating, burpees, and side planks. The body weight strength circuit training was performed at 60–75% HR_max_, with an RPE of 11–16. Capillary blood glucose concentration was measured directly after completion.

At 12:00, participants were served a standardized low-protein lunch. They could eat as much as they wanted from the food items provided: white bread (Loff; First Price, Norway), apple, jam (Bringebærsyltetøy; First Price, Norway), and vegan cheese (Go’Vegan Original; Synnøve, Norway). The calculated macronutrient content of this meal was carbohydrate 1.31 ± 0.10 g·kg^−1^, protein 0.21 ± 0.01 g·kg^−1^, fat 0.51 ± 0.06 g·kg^−1^, calorie intake 807.1 ± 52.1 kcal.

At 14:00, capillary blood glucose concentration was measured. Participants started 1 h of cardio endurance training. They could choose to distribute their time between cycling, running, and rowing on a rowing machine. The 1 h of cardio endurance training was performed at 60–80% HR_max_, with an RPE of 9–14. Capillary blood glucose concentration was measured directly after the cardio endurance training.

At 15:00, participants started 1 hour of cycling to exhaustion on a stationary spinning bike (M3i Indoor Bike, Keiser, California, United States). The cycling protocol consisted of 10 min warm-up (50–60% HR_max_); 3×3 min at 80% HR_max_ interspersed with 3 min at 60–70% HR_max_; 3×30 s at 90% HR_max_ interspersed with 30 s at 70% HR_max_; 3×2 min at 90% HR_max_ interspersed with 2 min at 70% HR_max_; 3×30 s at 90–95% HR_max_ interspersed with 30 s at 70–80% HR_max_; 5 min cool-down at 50–60% HR_max_. The final exercise block of cycling was performed to exhaustion, with a target heart rate of 90–95% HR_max_ and RPE of 18–20. Capillary blood glucose concentration was measured at the end of the cycling to exhaustion. The participants’ body weight was measured directly at the end of the cycling to exhaustion.

#### 2.4.2 Dietary intervention

Participants were asked to record their breakfast, lunch, snacks, dinner, and evening meal, as well as all drinks of the trial day and breakfast the next day. A dietary registration form was distributed to the participants for this purpose, and participants were encouraged to take photos of their food intake to improve the reproducibility of intake quantities. They were then asked to consume the same food and drinks on the second trial day. They had to check off on the dietary registration form the items ingested. Any differences in nutritional intake during the second visit had to be reported and accurately described on the dietary registration form. No participant reported a difference in dietary intake. The total calorie intake, carbohydrate, protein, and fat intakes were calculated using the Kostholdsplanleggeren program (Norwegian Directorate of Health, Norwegian Food Safety Authority, Norway). Table 2 presents the dietary intake during the trial days.

**Table 2:** Energy intake and content of macronutrients for the different standardized meals and supplements during the protocol.

	Energy intake (kcal)	Carbohydrate intake (g⋅kg^−1^)	Protein intake (g⋅kg^−1^)	Fat intake (g⋅kg^−1^)
Breakfast	570.3 ± 81.3	1.18 ± 0.20	0.23 ± 0.02	0.22 ± 0.04
Lunch	807.1 ± 52.1	1.31 ± 0.10	0.21 ± 0.01	0.51 ± 0.06
Snacks (during EX)	421.9 ± 51.8	1.09 ± 0.14	0.07 ± 0.01	0.10 ± 0.01
Dinner	776.7 ± 117.6	0.81 ± 0.11	0.62 ± 0.11	0.48 ± 0.13
Evening meal	626.7 ± 138.7	1.06 ± 0.21	0.32 ± 0.10	0.31 ± 0.08
Breakfast the next day	531.4 ± 73.7	1.03 ± 0.14	0.22 ± 0.02	0.23 ± 0.05
Total CON	3312.2 ± 284.2	5.39 ± 0.44	1.60 ± 0.13	1.75 ± 0.22
Total EX	3734.1 ± 301.6	6.48 ± 0.48	1.67 ± 0.14	1.85 ± 0.22

The total macronutrient amount in CON and EX is calculated from all food intake during the urine collection period.

Water intake was *ad libitum* during both trial days, and the volume was recorded.

During EX, participants were allowed to eat the high-carbohydrate snacks provided: banana, cereal bar (Mellombar; Eldorado, Norway), and milk chocolate (Kokesjokolade; Eldorado, Norway). They could also drink as much of a carbohydrate drink as they wanted. The drink was prepared as a 10% solution containing 50% glucose (GPR RECTAPUR®, VWR Chemicals, Radnor, Pennsylvania, USA) and 50% maltodextrin (Dextri-maltose®, MP Biomedicals, Santa Ana, California, USA), and flavored with 100 g·L^−1^ of drink flavoring (Fun light, Stabburet, Norway). These snacks and drinks were given to participants to ensure the same energy balance during the two trial days. The calculated macronutrient content of the snacks was carbohydrate 1.09 ± 0.14 g·kg^−1^, protein 0.07 ± 0.01 g·kg^−1^, fat 0.10 ± 0.01 g·kg^−1^, calorie intake 421.9 ± 51.8 kcal.

Nitrogen intake was calculated assuming a nitrogen-protein constant of 6.25 (39).

#### 2.4.3 Individual energy expenditure

The individual energy expenditure during exercise in EX was calculated from the heart rate data measured during the exercise. The oxygen uptake was then calculated from the heart rate data using the linear regression between the heart rate and the oxygen uptake established during the incremental test on the screening day. The energy expenditure was then calculated as oxygen uptake (L·min^−1^) × time exercise (min) × 20.15 (kJ·L^−1^) (40).

The resting energy expenditure in CON was calculated as (41):


$$\begin{array}{c} \mathrm{Resting}\;\mathrm{energy}\;\mathrm{expenditure}=9.99\;\times \;\mathrm{body}\;\mathrm{weight}+6.25\;\times \;\mathrm{height}\\ \;-4.92\;\times \;\mathrm{age}+166\;\times \;\mathrm{sex}\;\\ \;\left(\mathrm{males},\;1;\;\mathrm{females},\;0\right)-161 \end{array}$$

### 2.5 Urinary nitrogen

All urine was collected in plastic containers in three consecutive batches: morning urine on the trial day (0:00 to 8:00; 8 h), the rest of the day on the trial day (8:00 to 24:00; 16 h), and the morning urine on the next day (0:00 to 8:00; 8 h). The urine volume was measured for each period, and one 15-mL sample and two 2-mL samples were frozen at −20°C for analysis. Urinary nitrogen was measured using the Kjeldahl method (42) and corrected by urine density.

### 2.6 Sweat rate

The sweat rate during the exercise period was calculated using a modified equation (24, 43):


$$\begin{array}{c} \mathrm{Sweat}\;\mathrm{rate}\;\left(L\right)=BWi-BWf+\mathrm{WatIn}-ARW\;\mathrm{at}\;\mathrm{Loss}\\ -\mathrm{UrVol}\;+\;\mathrm{WFoodIn}\;-\;\left(EE\right)/7700 \end{array}$$

With BWi the initial body weight (kg), BWf the final body weight (kg), WatIn the water intake (L), ARW at Loss the assumed respiratory water loss (L), UrVol the urine volume (L), WFoodIn the weight of food intake (kg) and EE the energy expenditure during the exercise period (kcal).

Sweat volume and mass were considered equivalent (i.e., 1 mL = 1 g) and expressed as a total and hourly rate.

### 2.7 3-Methylhistidine in urine

The urine from the day of the trial day (8:00 to 24:00; 16 h) and the morning urine from the next day (0:00 to 8:00; 8 h) were mixed in proportions relative to the volume of urine during these periods. The concentration of urinary 3-MH was then quantified in these pooled urine samples using an ELISA kit (BioSite ELISA, Nordic BioSite, Norway). The quantity of 3-MH excreted during these 24 h was then calculated by multiplying the measured 3-MH concentration by the urine volume.

### 2.8 Statistics

The results were analyzed using Prism 9 (GraphPad Software, LLC, San Diego, California, United States). Two-way ANOVA (time x condition) with repeated measurements was conducted to analyze the data. After identifying a significant effect, *post hoc* analyses were performed with Bonferroni corrections. When some data were missing, mixed-effect analyses were used. The significance level was set to *p* ≤ 0.05. Statistical trends are defined as *p*-values between 0.05 and 0.10. Data are presented as mean ± SEM in text, figures, and tables.

## 3 Results

### 3.1 Energy expenditure

A description of the energy intake during the study is shown in Table 2. The total energy intake in CON was 2780.8 ± 242.1 kcal during the intervention day and 3312.2 ± 284.2 kcal during the urine collection period (including breakfast the next day). Therefore, the participants had a positive energy balance in CON during the intervention day of 1092.6 ± 228.2 kcal.

In EX, the total energy expenditure during the 5-h exercise period was 3289.9 ± 188.8 kcal or 44.6 ± 2.2 kcal·kg^−1^, meaning that during EX, participants had an additional energy expenditure of 2938.2 ± 182.5 kcal compared to the resting energy expenditure measured in CON. The participants were allowed to eat additional almost protein-free snacks during EX to compensate for this additional energy expenditure. They consumed 421.9 ± 51.8 kcal extra, thus, participants had a negative energy balance during EX of −1423.6 ± 219.5 kcal.

### 3.2 Urinary nitrogen excretion

**Figure 2:**
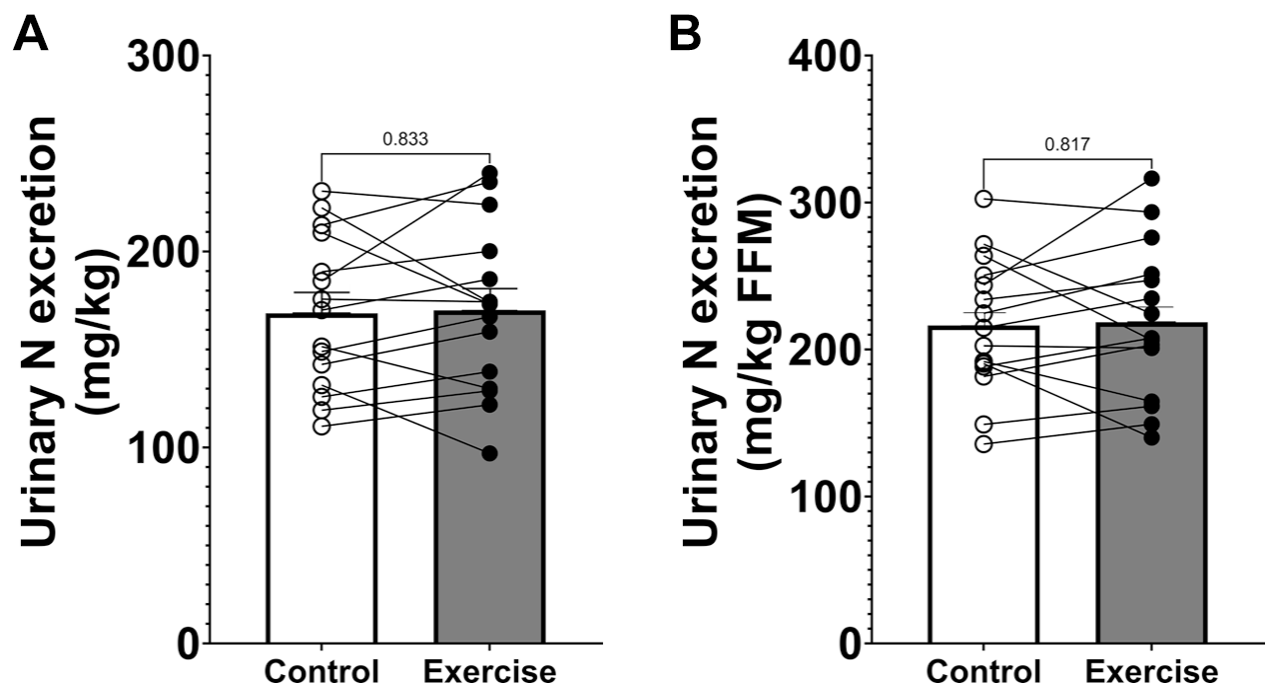
Urinary nitrogen excretion. **(A)** Urinary nitrogen excretion normalized by body weight. **(B)** Urinary nitrogen excretion normalized by fat-free mass. Values are means ± SEM.

Urinary nitrogen excretion during the 24-h study period was 12.45 ± 0.88 g (168.5 ± 10.1 mg·kg^−1^) in CON and 12.61 ± 0.98 g (170.0 ± 11.0 mg·kg^−1^) in EX (Figure 2A) and did not differ significantly between trial days (*p* = 0.764). Urinary nitrogen excretion was also not significantly different when normalized by fat-free mass (Figure 2B).

### 3.3 Sweat rate

The sweat rate was 2.55 ± 0.25 L during EX (Table 3). This represents an hourly sweating rate of 0.32 ± 0.03 L·h^−1^. During CON, the sweat rate was 0.14 ± 0.15 L, representing an hourly sweating rate of 0.02 ± 0.02 L·h^−1^.

**Table 3:** Sweat rate during the two conditions.

Condition	EX		CON	
From 8:00 to 16:00				
Initial body weight (kg)	73.8 ± 1.9		74.1 ± 2.0	
Final body weight (kg)	73.3 ± 2.0		74.2 ± 2.0	
Energy expenditure (kcal)	3,501 ± 193		563 ± 13	
	Intake	Loss	Intake	Loss
Water intake (L)	3.69 ± 0.39		1.45 ± 0.17	
Urine (L)		0.79 ± 0.17		1.13 ± 0.13
Respiratory water loss (L)		0.36 ± 0.02		0.05 ± 0.00
Sweat (L)		2.55 ± 0.25		0.14 ± 0.15

### 3.4 Urinary 3-methylhistidine excretion

**Figure 3:**
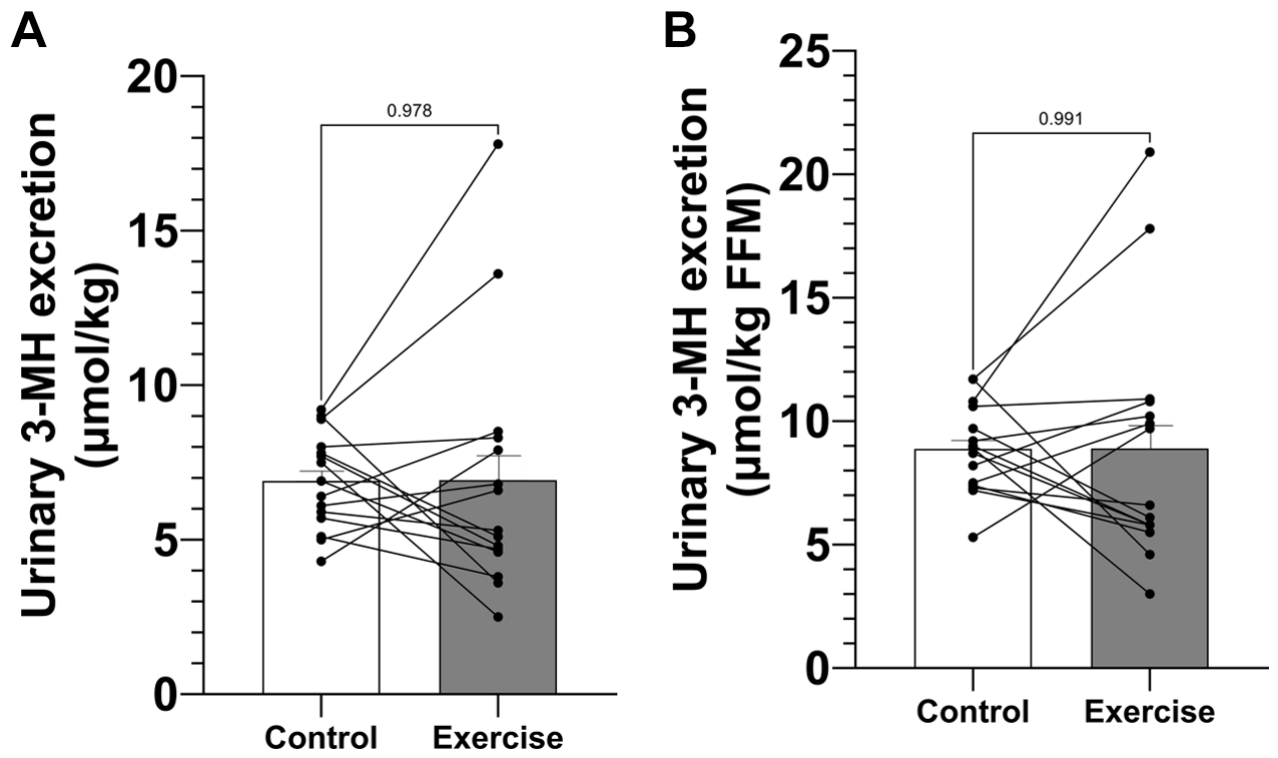
Urinary 3-MH excretion. **(A)** Urinary 3-MH excretion normalized by body weight. **(B)** Urinary 3-MH excretion normalized by fat-free mass. Values are means ± SEM.

Urinary 3-MH excretion during the 24-h study period was 508.7 ± 33.8 μmol (6.91 ± 0.40 μmol·kg^−1^) in CON and 513.0 ± 80.2 μmol (6.91 ± 1.05 μmol·kg^−1^) in EX (Figure 3A) and was not significantly different (*p* = 0.953). When normalized by fat-free mass, urinary 3-MH excretion did still not differ between trial days: 8.87 ± 0.47 μmol·kg^−1^ FFM in CON and 8.89 ± 1.27 μmol·kg^−1^ FFM in EX (*p* = 0.991) (Figure 3B).

### 3.5 Correlations

**Figure 4:**
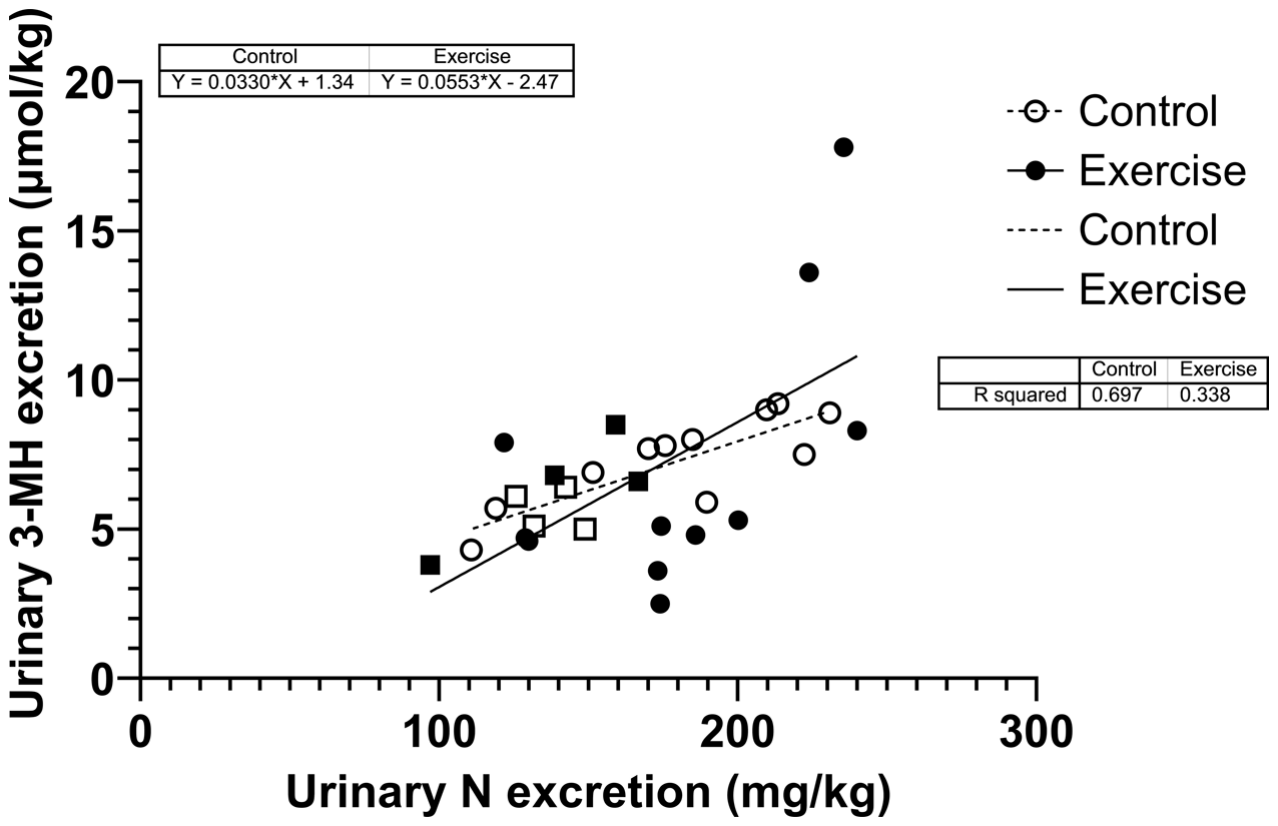
Correlation between urinary nitrogen and 3-MH excretions. Square dots are female participants; circular dots are male participants.

Urinary nitrogen excretion was significantly correlated with urinary 3-MH excretion but higher in CON (*r*^2^ = 0.697; *p* < 0.001) than in EX (*r*^2^ = 0.338; *p* = 0,023) (Figure 4). The two slopes were not significantly different (*p* = 0.343).

**Figure 5:**
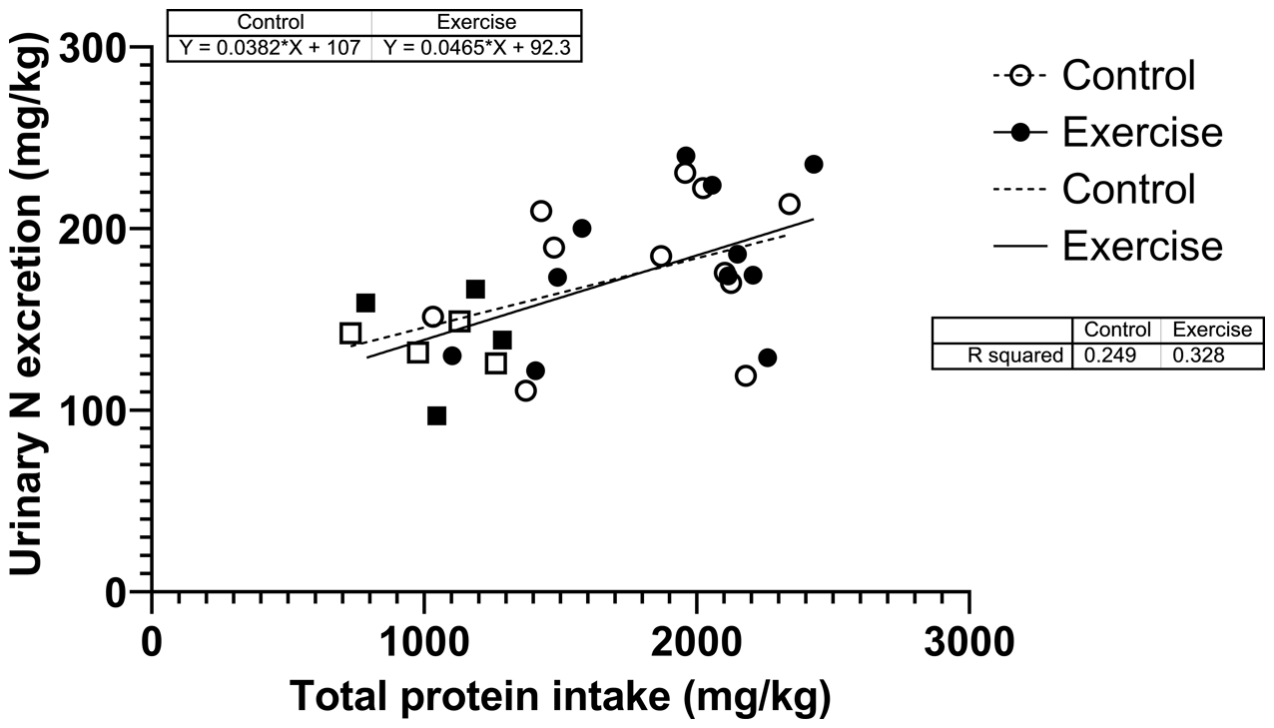
Correlation between urinary nitrogen excretion and total protein intake during the urine collection period. Square dots are female participants; circular dots are male participants.

Urinary nitrogen excretion was also correlated with total protein intake over the study period in CON (*r^2^* = 0.249; *p* = 0.058) and in EX (*r*^2^ = 0.328; *p* = 0.026) (Figure 5). The two slopes were not significantly different (*p* = 0.752).

### 3.6 Blood glucose concentration

**Figure 6:**
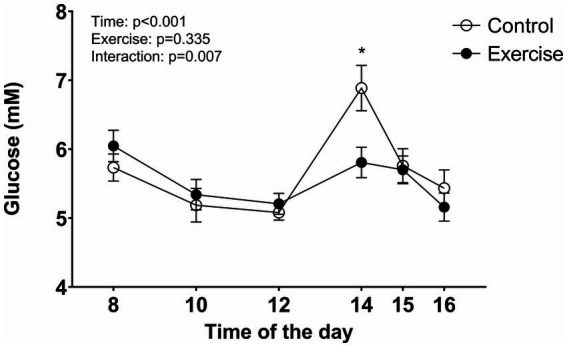
Blood glucose concentration during the test days. Values are means ± SEM.

The blood glucose concentration decreased during EX and CON with no difference up to the lunch break at 12:00 (Figure 6). However, at 14:00, 90 min after lunch completion, the glucose concentration was significantly elevated in CON compared to EX (*p* = 0.001). After that, the blood glucose concentration decreased with no difference between the conditions.

## 4 Discussion

Urinary nitrogen excretion during the 24-h study period did not significantly differ between EX and CON.

### 4.1 Reasons for the lack of difference observed in urinary nitrogen excretion in the present study

Disparities in urinary nitrogen excretion have been reported during exercise compared to a control condition. Most of the cited studies in Supplementary Table S1 reported increased urinary nitrogen excretion during or after exercise (3, 5–12, 14–17, 44–51), not always being significant (18, 52–56). However, because of the limitations, which included the nitrogen excretion quantification method, no standardized diet, no control trial in the same subjects, small sample sizes, and no specific energy expenditure calculation during the work/exercise periods, no clear conclusion has been reached on the effect of exercise on urinary nitrogen excretion. Newer studies have also tended to indicate that exercise increases urinary nitrogen excretion, but the absence of standardized conditions still limits the conclusions reached.

#### 4.1.1 The effect of energy balance

In similar conditions to the present study, when participants were in negative energy balance with exercise, Todd et al. (57) and Butterfield and Calloway (58) found that urinary nitrogen excretion increased with exercise but non-significantly: there was an average 4% increase, ranging from −3 to 9%. This is in the order of magnitude of the 1% increase in the present study. However, at a fixed energy balance (when the extra energy expenditure of training was compensated by additional energy intake), exercise decreased urinary nitrogen excretion by 7% on average, ranging from −11 to 2% (57, 58). It is well established that increasing energy intake decreases nitrogen excretion at fixed nitrogen intake (13, 59). This shows that the effect of exercise on urinary nitrogen excretion can be caused by a decreased energy balance with exercise. This agrees with studies reporting increased excretion with exercise when energy intake was insufficient (3, 47, 55, 56). In the present study, participants were allowed to ingest almost protein-free snacks in EX in an attempt to have a fixed energy balance between the conditions, although the energy balance was still negative in EX. However, the ingestion of snacks in EX could have reduced the effect of exercise on urinary nitrogen excretion, leading to the non-significant difference between conditions.

To our knowledge, the mechanism for decreased urinary nitrogen excretion with exercise while the participants are in energy balance has not yet been established. However, some studies showed that low glycogen levels and low carbohydrate availability can increase nitrogen excretion (24, 35).

### 4.2 Sweat nitrogen excretion

Urine is not the only way for the body to excrete nitrogen. Nitrogen can also be excreted in sweat, feces, or other miscellaneous routes [nails, hair, tooth brushing (21)]. Nitrogen excretion through these different compartments is connected. Some studies have previously reported a link between urinary and sweat nitrogen excretion: urinary nitrogen excretion decreased in proportion when sweat nitrogen losses increased (19, 20), as in the studies commented on previously (57, 58). This could also explain why the urinary nitrogen excretion was not significantly different between EX and CON. In similar conditions to the present study, sweat nitrogen excretion increased by 22% on average, ranging from −20 to 58% when the participants exercised while in energy balance (57, 58).

In the present study, we calculated the sweat rate and reported an hourly sweating rate of 0.32 ± 0.03 L·h^−1^ during EX. This agrees with sweat rates during exercise reported in similar ambient conditions (43). However, we did not measure the sweat nitrogen concentration. Using previously published data, we can estimate this concentration. For this purpose, we used the relationship between sweat nitrogen concentration and nitrogen intake established during exercise performed mid-day under similar thermal conditions (20, 25). We used this estimation because sweat nitrogen concentration has been shown to differ with time of day, temperature, and dietary nitrogen intake, with this last factor having the most significant impact on sweat nitrogen concentration. Based on the regression between sweat nitrogen concentration and protein intake established during exercise conditions (20), estimated nitrogen excretion through sweating during exercise was ≈1.3 g in EX. The estimated average sweat nitrogen concentration was 0.5 mg/mL, consistent with previous studies (19, 21, 23, 24).

In addition, resting sweat nitrogen excretion can be estimated from the relationship between resting sweat nitrogen excretion and nitrogen intake (21): resting sweat nitrogen excretion (mg·d^−1^) = 4.8022 x nitrogen intake (g·d^−1^) + 104. This led to a resting sweat nitrogen excretion of ≈0.2 g in CON and EX, with no significant difference between conditions. Finally, fecal nitrogen loss can be estimated from previous studies at 12.41 mg·kg^−1^·d^−1^ and miscellaneous nitrogen losses at 1.77 mg·kg^−1^·d^−1^ (25). The total nitrogen excretion can then be calculated as the sum of the urinary, sweat, fecal, and miscellaneous nitrogen excretions.

### 4.3 Sweat nitrogen excretion should be included in future studies

Including or excluding sweat nitrogen excretion can dramatically change a study’s conclusions. For example, the nitrogen balance technique calculates the difference between nitrogen intake and loss. A positive nitrogen balance shows that the human body stores more nitrogen than it loses: it is in an anabolic state. Similarly, a negative nitrogen balance indicates a catabolic state. Thus, the sign of the nitrogen balance is crucial for any interpretation.

Gontzea et al. (19) conducted a study in which six males cycled for 2 h daily in laboratory conditions, and all nitrogen excretion pathways were measured. In these well-controlled conditions, with all food intake provided, the authors studied the effect of including nitrogen loss through sweat on nitrogen balance. If sweat nitrogen losses were neglected, 1 in 6 participants had a positive nitrogen balance (average − 0.827 g·d^−1^) when exercising with protein intakes between 65 and 75 g. When sweat nitrogen losses were considered, all 6 participants had a negative nitrogen balance (average − 1.721 g·d^−1^). Similarly, when exercising with a protein intake of between 105 and 115 g, if sweat nitrogen losses were neglected, 4 in 6 participants had a positive nitrogen balance (average 0.676 g·d^−1^). When sweat nitrogen losses were considered, only 2 in 6 participants had a positive nitrogen balance (average − 0.371 g·d^−1^). Based on these results, it could be concluded that the protein intake was enough to induce a positive nitrogen balance in the last case if sweat nitrogen losses were neglected. Nevertheless, this conclusion would have been erroneous. Similarly, in another study, ignoring sweat nitrogen losses resulted in a positive nitrogen balance (average 0.62 g·d^−1^) (60). When sweat nitrogen losses were included, the nitrogen balance was negative (average − 2.01 g·d^−1^), changing the conclusion of this intervention.

In the present study, the nitrogen balance was positive in the two conditions both when sweat nitrogen losses were considered (CON, 70.8 ± 18.3 mg·kg^−1^; EX, 63.0 ± 16.8 mg·kg^−1^) and when they were ignored and only urinary nitrogen losses were considered (CON, 73.4 ± 18.4 mg·kg^−1^; EX, 83.2 ± 17.8 mg·kg^−1^). Thus, including sweat nitrogen losses would not have changed the interpretation. However, including them would have considerably increased the total nitrogen excretion in EX. Total nitrogen excretion during the 24-h study period would have been 13.69 ± 0.90 g (185.3 ± 10.2 mg·kg^−1^) in CON and 15.15 ± 1.14 g (204.3 ± 12.6 mg·kg^−1^) in EX, leading to a significant effect of exercise (*p* = 0.033). It means that proteins contributed to 1.3% of energy expenditure during exercise. It is, therefore, crucial to include sweat nitrogen losses when quantifying nitrogen excretion. Studies without direct measurements should estimate these losses.

The magnitude of the effect of exercise on total nitrogen excretion can be compared to results from previous studies (Supplementary Table S2) (19, 57, 58, 61–63). Exercise was reported to increase total nitrogen excretion in four of these six studies, with an average increase of 0.23 g·d^−1^. Of these six studies, the two reporting decreased total nitrogen excretion with exercise also showed an inappropriate nitrogen intake before the intervention days, leading to negative nitrogen balance at baseline (62, 63). It is well-established that nitrogen excretion increases with nitrogen intake (13), which is consistent with our data. Therefore, it is expected that some differences in the amount of nitrogen excretion should arise. However, our estimation of 1.5 g·d^−1^ was in the range of variation of previously published studies, which showed levels between −1.8 and 2.6 g·d^−1^ (19, 57, 58, 61–63). Such an increase in total nitrogen excretion during EX would mean that exercise increased protein catabolism. However, this does not indicate the location of this degradation.

### 4.4 Urinary 3-methylhistidine excretion

Urinary 3-MH excretion during the 24-h study period was not significantly different between EX and CON. This would mean no significant increase in skeletal myofibrillar protein catabolism with exercise. Previous studies measuring urinary 3-MH excretion during exercise have reported conflicting results. Most of these studies did not note any significant difference in urinary 3-MH excretion with exercise (17, 64–71), while some reported an increase (72) and other a decrease (73, 74). These studies consisted of acute endurance exercises, mostly of shorter duration than this study. Studies similar in duration to the present study did not find a difference in urinary 3-MH excretion (64, 65, 67), apart from one showing a decrease (74). This latter study included urinary 3-MH excretion measured at different periods on the day of exercise. In this study, urinary 3-MH excretion was not different from the control day, before and after exercise. However, the drop in excretion during exercise meant that the total excretion on the exercise day was significantly lower than on the control day. All this indicates that exercise does not increase urinary 3-MH excretion and therefore, does not affect the catabolism of skeletal myofibrillar proteins.

### 4.5 Practical considerations

#### 4.5.1 Daily variations of the urinary nitrogen excretion

The day-to-day variation in urinary nitrogen excretion in resting conditions varies from ≈4% (75) to ≈14% (76), with most studies reporting around 10% (77, 78). In exercise conditions, the day-to-day variation in urinary nitrogen excretion seems similar, with Gontzea et al. reporting ≈9% (19).

One strength of the present study is that it used a randomized, balanced, cross-over design. This design, combined with the same diet before and during both trial days, except for snacks in EX, should have reduced variation. Including 15 participants further reduced the influence of daily variations on the conclusion about the effect of exercise on urinary nitrogen excretion.

#### 4.5.2 Participants’ habitual protein intake

A potential weakness of the present study was that not all food was supplied during the intervention days. Participants received a standardized lunch. However, they were free to ingest the food of their choice during the rest of the intervention and only had to replicate this intake on the second trial day. This intentionally allowed participants to consume their habitual protein intake during the trial days. It has previously been established that, when changing from high to low protein intake, or vice versa, a few days are necessary before a nitrogen balance is regained: the amino acid oxidative capacity of the organism is determined by the habitual protein intake (79).

#### 4.5.3 Nitrogen excretion the day after an exercise period

Some studies suggest that increased nitrogen output continues after the exercise stops (53–55). Consequently, the increase in output may be more significant on the succeeding day (49). Similarly, urinary urea excretion decreased immediately after a 100-km run but increased the day after (67). In the present study, urinary nitrogen excretion was measured up to 8:00 the day after EX and CON, yet no significant difference was observable within this period. In addition, total production (excretion + retention) increased immediately after a 100-km run and decreased the day after (67). It is therefore unlikely that some differences appeared the day after that could have compensated for the effects of exercise.

## 5 Conclusion

Our findings suggest that exercise did not significantly impact urinary nitrogen excretion. It is possible that the effect of exercise on urinary nitrogen excretion was reduced due to the reduction of the energy deficit, which was achieved by consuming almost protein-free snacks during exercise. As the sweat rate was measured in the present study, the sweat nitrogen excretion could be estimated based on previously published sweat nitrogen concentrations. This enabled us to estimate that exercise led to a significant increase in total nitrogen excretion. However, the urinary 3-MH excretion, which is an indicator of myofibrillar protein breakdown, did not change significantly with exercise. We recommend that future studies include measurements of sweat nitrogen excretion or at least an estimate to determine the effect of exercise on nitrogen excretion accurately.

## Data availability statement

The raw data supporting the conclusions of this article will be made available by the authors, without undue reservation.

## Ethics statement

The studies involving humans were approved by the Norwegian School of Sport Sciences’ Ethics Committee (Application 190-170621) and the Norwegian Centre for Research Data (Reference number 473323). The studies were conducted in accordance with the local legislation and institutional requirements. The participants provided their written informed consent to participate in this study.

## Author contributions

MC: Conceptualization, Formal analysis, Investigation, Methodology, Writing – original draft, Writing – review & editing. MB: Formal analysis, Investigation, Writing – review & editing. SW: Formal analysis, Investigation, Writing – review & editing. BS: Conceptualization, Methodology, Supervision, Writing – review & editing. JJ: Conceptualization, Funding acquisition, Methodology, Supervision, Writing – review & editing.
