# Corrigendum: Effect of five hours of mixed exercise on urinary nitrogen excretion in healthy moderate-to-well-trained young adults

**DOI:** 10.3389/fnut.2024.1406641

**Published:** 2024-04-09

**Authors:** Matthieu Clauss, Meike Burkhardt, Sophie Wöber, Bjørn Steen Skålhegg, Jørgen Jensen

**Affiliations:** ^1^Department of Physical Performance, Norwegian School of Sport Sciences, Oslo, Norway; ^2^Department of Sport and Sport Science, University of Freiburg, Freiburg, Germany; ^3^Department of Nutrition, Division for Molecular Nutrition, University of Oslo, Oslo, Norway

**Keywords:** Urinary nitrogen excretion, sweat nitrogen excretion, protein metabolism, endurance exercise, 3-methylhistidine excretion

In the published article, there was an error. A piece of data is wrong.

A correction has been made to **4. Discussion**, *4.3 Sweat nitrogen excretion should be included in future studies*, Paragraph 3. This sentence previously stated:

“It means that proteins contributed to 0.3% of energy expenditure during exercise.”

The corrected sentence appears below:

“It means that proteins contributed to 1.3% of energy expenditure during exercise.”

The authors apologize for this error and state that this does not change the scientific conclusions of the article in any way. The original article has been updated.

