# Supplementary material for: Effect of five hours of mixed exercise on urinary nitrogen excretion in healthy moderate-to-well-trained young adults
Source: Front Nutr. 2024 Feb 21;11:1345922. doi: 10.3389/fnut.2024.1345922 (PMC10914964; doi:10.3389/fnut.2024.1345922)
Supplement: Supplementary file 1 [file Table_1.DOCX]

**Table S1. Studies reporting nitrogen excretion measured in urine during a control and an exercise day.**

| Reference | Type exercise | Number of participants | N intake  (g∙d^-1^) | N intake  (g∙d^-1^) | N excretion  (g∙d^-1^) | N excretion  (g∙d^-1^) | Variation N excretion  (g∙d^-1^) | N excretion measurement compartment |
| --- | --- | --- | --- | --- | --- | --- | --- | --- |
|  |  |  | Day(s) before | Exercise day(s) | Day(s) before | Exercise day(s) |  |  |
| (1) | Walking 62 miles | 1 | 22 | 13.2 # | 20.1 | 23.2 | +3.1 | Urine |
| (2) | 32km walking | 1 | n. r. | n. r. | 46.23 | 50.99 | +4.8 | Urine |
| (3) | Walking 65,5 miles in 24 hours | 1 | n. r. | n. r. | 17.75 | 38.92 | +21.2 | Urine |
| “ | Walking 180 miles in 2 days | 1 | n. r. | n. r. | 19.65 | 32.81 | +13.2 | Urine |
| “ | Walking 263 miles in 3 days | 1 | n. r. | n. r. | 15.22 | 34.84 | +19.6 | Urine |
| “ | Walking 450 miles in 6 days | 1 | n. r. | n. r. | 20.62 | 33.8 | +13.2 | Urine |
| (4) | Walking 317,5 miles in 5 days | 1 | n. r. | n. r. | 20.42 | 23.43 | +3.0 | Urine |
| (5) | Turning a crank, 12666 revolutions per day, 14 lbs. pressure | 6 | 13.21 | 13.21 | 16.09 | 16.55 | +0.5 | Urine |
| (6) | Walking 5 h, strength 1 h | 1 | 12.48 | 12.76 | 15.34 | 16.31 | +1.0 | Urine |
| (7) | Walking 20 km 1600 m D+ in 12 hours | 1 | n. r. | n. r. | 13.24 | 18.61 | +5.4 | Urine |
| “ | Walking 12 km 1300 m D+ in 5 hours | 1 | n. r. | n. r. | 14.07 | 15.13 | +1.1 | Urine |
| (8) | Walking 1137,7 m D+ | 1 | 15.87 | 15.87 | 17.08 | 18.29 | +1.2 | Urine |
| “ | Walking 2403,79 m D+ | 1 | 15.87 | 15.87 | 17.08 | 18 | +0.9 | Urine |
| (9) | Cycling 86 miles hilly | 1 | 26.3 | 26.3 | 16.93 | 21.42 | +4.5 | Urine |
| (9) | Walking 13 miles, riding 12 miles, digging 1 h 40 min, and ascending a hill of the height of 800 feet | 1 | 18.8 | 18.8 | 13.39 | 13.8 | +0.4 | Urine |
| (9) | Cycling 50 miles, walking 5 miles and digging for 2 h | 1 | 17.8 | 17.8 | 13.86 | 14.46 | +0.6 | Urine |
| (10) | Ergostat 17 000 kgm of work every day in 18 days | 1 | 13.21 | 19.96 # | 12.53 | 18.485 | +6.0 | Urine |
| (11) | Cycling 6-day race (1822,6 miles) | 3 | 29.1 | 29.1 | 19.3 | 36.2 | +16.9 | Urine |
| (12) | Walking | 1 | n. r. | n. r. | 21.44 | 22.21 | +0.8 | Urine |
| (13) | Walking 60 km | 1 | 1.01 | 1.01 | 2.93 | 3.89 | +1.0 | Urine |
| (13) | Walking 60 km | 1 | 1.01 | 1.01 | 3.07 | 3.77 | +0.7 | Urine |
| (13) | Walking 12 km and 30 min swimming | 1 | 40.74 | 40.74 | 26.99 | 39.38 | +12.4 | Urine |
| “ | Walking 33 km | 1 | 40.74 | 40.74 | 38.63 | 38.4 | -0.2 | Urine |
| “ | Walking 60 km | 1 | 40.74 | 40.74 | 38.98 | 38.7 | -0.3 | Urine |
| (13) | Walking 1 h 30 min swimming | 1 | 33.83 | 33.83 | 32.79 | 33.53 | +0.7 | Urine |
| “ | Walking 60 km | 1 | 33.83 | 33.83 | 33.6 | 37.79 | +4.2 | Urine |
| (13) | Walking 33 km | 1 | 21.4 | 21.4 | 22.26 | 19.66 | -2.6 | Urine |
| “ | Walking 33 km | 1 | 21.4 | 21.4 | 19.55 | 19.71 | +0.2 | Urine |
| (14) | Various exercise 6 h | 1 | 8.96 | 8.96 | 11.08 | 11.68 | +0.6 | Urine |
| (14) | Cycling 40 miles hilly | 1 | 4.83 | 4.83 | 8.81 | 8.06 | -0.8 | Urine |
| (14) | Cycling 40 miles hilly | 1 | 4.83 | 4.83 | 6.74 | 6.92 | +0.2 | Urine |
| (15) | 6,5 h normal laboratory work | 1 | n. r. | n. r. | 8.18 | 9.12 | +0.9 | Urine |
| “ | 1,5 h normal laboratory work + 5 h work 13 500 kgm per hour | 1 | n. r. | n. r. | 8.18 | 9.65 | +1.5 | Urine |
| (16) | 6,5 h normal laboratory work | 1 | n. r. | n. r. | 8.01 | 9.015 | +1.0 | Urine |
| “ | Light muscular work (67 500 kgm in 5 hours) | 1 | n. r. | n. r. | 8.01 | 9.465 | +1.5 | Urine |
| “ | Severe muscular work (100 000 kgm in 5 hours) | 1 | n. r. | n. r. | 8.01 | 10.25 | +2.2 | Urine |
| (17) | Cycling 1 h 30 at 46% VO_2max_ | 8 | 9.6 | 9.6 | 10.73 | 14.63 | +3.9 | Urine |
| (18) | Running 15.5 km at 65% VO_2max_ | 6 | 20.9 | 20.9 | 16.47 | 22.35 | +5.9 | Urine |
| (19) | Cycling 60 min at 75% VO_2max_ + TTE 85% VO_2max_ / Low CHO diet | 7 | 18.6 | 18.6 | 12.24 | 14.00 | +1.8 | Urine |
| “ | Cycling 60 min at 75% VO_2max_ + TTE 85% VO_2max_ / High CHO diet | 7 | 17.2 | 17.2 | 12.12 | 15.76 | +3.6 | Urine |
| (20) | Cycling 90 min at 65% VO_2max_ | 8 | 23.0 | 23.0 | 10.71 | 11.41 | +0.7 | Urine |
| (21) | Cycling 90 min at 70% VO_2max_ | 10 | 19.7 | 19.7 | 15.0 | 16.0 | +1.0 | Urine |
| “ | Cycling 90 min at 70% VO_2max_ | 10 | 19.0 | 19.0 | 16.0 | 19.0 | +3.0 | Urine |

n. r. not reported. # difference in nitrogen intake between control day and exercise day.

1. Flint. *New York Med Jour* (1870) 13:653.

2. Schenk. Über den Einfluss der Muskelarbeit auf die Eiweisszersetzung im menschlichen Organismus [About the influence of muscle work on protein breakdown in the human organism]. *Archiv fur Experimentelle Pathologie und Pharmakologie* (1874) II:21.

3. Pavy FW. The effect of prolonged muscular exercise upon the urine in relation to the source of muscular power. *The Lancet* (1876) 108:815–818. doi: 10.1016/S0140-6736(02)49561-X

4. Pavy FW. The effect of prolonged muscular exercise upon the urine in relation to the source of muscular power. *The Lancet* (1876) 108:887–889. doi: 10.1016/S0140-6736(02)31787-2

5. Brietzcke H. Urea and its relation to muscular force. *Br Foreign Med Chir Rev* (1877) 60:190–200.

6. Hirschfeld F. Beiträge zur Ernährungslehre des Menschen [Contributions to human nutrition]. *Archiv f pathol Anat* (1888) 114:301–340. doi: 10.1007/BF01882632

7. Argutinsky P. Muskelarbeit und Stickstoffumsatz [Muscle work and nitrogen turnover]. *Pflüger, Arch* (1890) 46:552–580. doi: 10.1007/BF01789543

8. Krummacher O. Über den Einfluss der Muskelarbeit auf die Eiweisszersetzung bei gleicher Nahrung [About the influence of muscle work on protein breakdown with the same food]. *Pflüger, Arch* (1890) 47:454–468. doi: 10.1007/BF01789825

9. Dunlop JC, Paton DN, Stockman R, Maccadam I. On the influence of muscular exercise, sweating, and massage, on the metabolism. *J Physiol* (1897) 22:68–91.

10. Bornstein K. Eiweissmast und Muskelarbeit [Protein pole and muscle work]. *Pflüger, Arch* (1901) 83:540–556. doi: 10.1007/BF01746508

11. Atwater, Sherman. The effect of severe and prolonged muscular work on food consumption, digestion, and metabolism. *US Department Agriculture Bulletin* (1901)98.

12. Kaup. *Zeitschrift für Biologie* (1902) XLIII:221.

13. Kocher RA. Über die Große des Eiweißzerfalls bei Fieber und bei Arbeitsleistung [About the extent of protein breakdown during fever and during work]. *Deutsches Archiv für klinische Medizin* (1914) CXV:82.

14. Boyd J. A contribution to the metabolism of creatine. (1914).

15. Campbell JA, Webster TA. Day and night urine during complete rest, laboratory routine, light muscular work and oxygen administration. *Biochemical Journal* (1921) 15:660–664. doi: 10.1042/bj0150660

16. Campbell JA, Webster TA. Effect of severe muscular work on composition of the urine. *Biochemical Journal* (1922) 16:106–110. doi: 10.1042/bj0160106

17. Calles-Escandon J, Cunningham JJ, Snyder P, Jacob R, Huszar G, Loke J, Felig P. Influence of exercise on urea, creatinine, and 3-methylhistidine excretion in normal human subjects. *Am J Physiol* (1984) 246:E334-338. doi: 10.1152/ajpendo.1984.246.4.E334

18. Tarnopolsky LJ, MacDougall JD, Atkinson SA, Tarnopolsky MA, Sutton JR. Gender differences in substrate for endurance exercise. *J Appl Physiol* (1990) 68:302–308. doi: 10.1152/jappl.1990.68.1.302

19. Tarnopolsky MA, Atkinson SA, Phillips SM, MacDougall JD. Carbohydrate loading and metabolism during exercise in men and women. *Journal of Applied Physiology* (1995) 78:1360–1368. doi: 10.1152/jappl.1995.78.4.1360

20. Tarnopolsky MA, Bosman M, Macdonald JR, Vandeputte D, Martin J, Roy BD. Postexercise protein-carbohydrate and carbohydrate supplements increase muscle glycogen in men and women. *J Appl Physiol (1985)* (1997) 83:1877–1883. doi: 10.1152/jappl.1997.83.6.1877

21. Broad EM, Maughan RJ, Galloway SDR. Carbohydrate, protein, and fat metabolism during exercise after oral carnitine supplementation in humans. *Int J Sport Nutr Exerc Metab* (2008) 18:567–584. doi: 10.1123/ijsnem.18.6.567
