# Supplementary material for: Effect of five hours of mixed exercise on urinary nitrogen excretion in healthy moderate-to-well-trained young adults
Source: Front Nutr. 2024 Feb 21;11:1345922. doi: 10.3389/fnut.2024.1345922 (PMC10914964; doi:10.3389/fnut.2024.1345922)
Supplement: Supplementary file 2 [file Table_2.DOCX]

**Table S2. Studies reporting nitrogen excretion measured in urine, sweat and feces during a control and an exercise day.**

| Reference | Type exercise | Number of participants | N intake  (g∙d^-1^) | N intake  (g∙d^-1^) | N excretion  (g∙d^-1^) | N excretion  (g∙d^-1^) | Variation N excretion  (g∙d^-1^) | N excretion measurement compartment |
| --- | --- | --- | --- | --- | --- | --- | --- | --- |
|  |  |  | Day(s) before | Exercise day(s) | Day(s) before | Exercise day(s) |  |  |
| (1) | Cycling in 20 min periods (113 min) | 30 | 11.63 | 11.63 | 11.17 | 13.23 | +2.1 | Urine + sweat + feces |
| (2) | 1h treadmill walking and 1h cycling | 6 | 6.6 | 6.6 | 6.3 | 6.6 | +0.4 | Urine + sweat + feces |
| (3) | 1h treadmill walking and 1h cycling / Very low protein diet | 6 | 7.8 | 7.8 | 7.1 | 7.2 | +0.1 | Urine + sweat + feces |
| “ | 1h treadmill walking and 1h cycling / Very low protein diet | 6 | 7.8 | 7.8 | 7.0 | 7.4 | +0.3 | Urine + sweat + feces |
| “ | 1h treadmill walking and 1h cycling / Low protein diet | 6 | 9.9 | 9.9 | 9.0 | 9.2 | +0.2 | Urine + sweat + feces |
| “ | 1h treadmill walking and 1h cycling / Low protein diet | 6 | 9.9 | 9.9 | 8.2 | 8.5 | +0.3 | Urine + sweat + feces |
| (4) | Cycling 90 min at 65% VO_2max_ | 6 | 9.6 | 9.6 | 6.5 | 7.8 | +1.3 | Urine + sweat + feces |
| (5) | 1,000 kcal cycling or running exercise (50–65% VO_2peak_) in 15-min bouts | 8 | 11.6 | 11.5 # | 13.0 | 11.5 | -1.4 | Urine + sweat + feces |
| “ | 1,000 kcal cycling or running exercise (50–65% VO_2peak_) in 15-min bouts | 7 | 11.6 | 11.8 # | 10.9 | 13.5 | +2.6 | Urine + sweat + feces |
| “ | 1,000 kcal cycling or running exercise (50–65% VO_2peak_) in 15-min bouts | 7 | 24.0 | 23.9 # | 25.8 | 24.4 | -1.4 | Urine + sweat + feces |
| (6) | 1,000 kcal cycling or running exercise (50–65% VO_2peak_) in 15-min bouts | 5 | 11.9 | 11.7 # | 11.6 | 11.6 | 0.0 | Urine + sweat + feces |
| “ | 1,000 kcal cycling or running exercise (50–65% VO_2peak_) in 15-min bouts | 6 | 11.9 | 11.8 # | 13.3 | 11.6 | -1.8 | Urine + sweat + feces |

n. r. not reported. # difference in nitrogen intake between control day and exercise day.

1. Gontzea I, Sutzesco P, Dumitrache S. Recherches concernant l’influence de l’activité musculaire sur le métabolisme azoté et sur le besoin en protéines de l’homme [Research concerning the influence of muscular activity on nitrogen metabolism and on the human protein requirement]. *Annales de la nutrition et de l’alimentation* (1968) 22:183–238.

2. Butterfield GE, Calloway DH. Physical activity improves protein utilization in young men. *BJN* (1984) 51:171. doi: 10.1079/BJN19840021

3. Todd KS, Butterfield GE, Calloway DH. Nitrogen balance in men with adequate and deficient energy intake at three levels of work. *J Nutr* (1984) 114:2107–2118. doi: 10.1093/jn/114.11.2107

4. Phillips SM, Atkinson SA, Tarnopolsky MA, MacDougall JD. Gender differences in leucine kinetics and nitrogen balance in endurance athletes. *J Appl Physiol* (1993) 75:2134–2141. doi: 10.1152/jappl.1993.75.5.2134

5. Pikosky MA, Smith TJ, Grediagin A, Castaneda-Sceppa C, Byerley L, Glickman EL, Young AJ. Increased protein maintains nitrogen balance during exercise-induced energy deficit. *Med Sci Sports Exerc* (2008) 40:505–512. doi: 10.1249/MSS.0b013e31815f6643

6. Smith TJ, Pikosky MA, Grediagin A, Castaneda-Sceppa C, Byerley LO, Glickman EL, Young AJ. Aerobic fitness does not modulate protein metabolism in response to increased exercise: a controlled trial. *Nutr Metab (Lond)* (2009) 6:28. doi: 10.1186/1743-7075-6-28
